# Persistent peristomal pain due to an exposed cutaneous nerve

**DOI:** 10.1016/j.jdcr.2026.02.013

**Published:** 2026-02-13

**Authors:** Iain Noel Encarnacion, Kaylin Beiter, Natasha Hakkal, Emily Hejazi, Paul Hernandez, Melissa Laughter, Shannon Nugent, Samir Thaker, Xiaowei Xu, Misha Rosenbach

**Affiliations:** a Eastern Virginia Medical School, Macon & Joan Brock Virginia Health Sciences at Old Dominion University, Norfolk, Virginia; b Department of Dermatology, Perelman School of Medicine at the University of Pennsylvania, Philadelphia, Pennsylvania; c Department of Medicine, Perelman School of Medicine at the University of Pennsylvania, Philadelphia, Pennsylvania; d Department of Colon and Rectal Surgery, Perelman School of Medicine at the University of Pennsylvania, Philadelphia, Pennsylvania; e Department of Anesthesiology and Critical Care, Perelman School of Medicine at the University of Pennsylvania, Philadelphia, Pennsylvania; f Department of Pathology and Laboratory Medicine, Perelman School of Medicine at the University of Pennsylvania, Philadelphia, Pennsylvania

**Keywords:** lidocaine, medical dermatology, neuropathic pain, peristomal pyoderma gangrenosum, pyoderma gangrenosum

## Case description

A 25-year-old woman with a history of ulcerative colitis treated with multiple abdominal surgeries complicated by peristomal pyoderma gangrenosum (PPG) presented with severe peristomal pain and ulceration. Treatment course was complicated; disease control was achieved via combination infliximab 10 mg/kg every 4 weeks, corticosteroids (topical triamcinolone 0.1% ointment, intralesional 10 mg/ml injections, and oral prednisone 30 mg daily with a gradual taper), and cyclosporine 125 mg tapering by 25 mg every 3 weeks, ultimately tapered to infliximab monotherapy.

**Fig 1:**
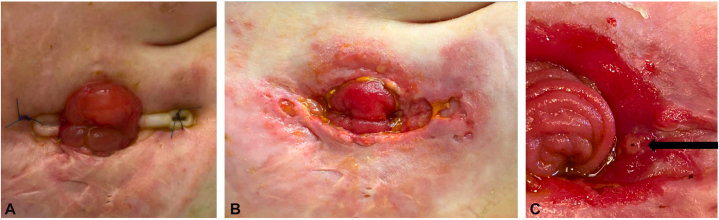
**A,** Concave ostomy site requiring a bolster, which placed additional focal pressure on the lateral aspects of the peristomal skin. **B,** Active peristomal pyoderma gangrenosum with cribriform scarring and erythematous ulcerations at the 3 and 9 o’clock positions. **C,** Peristomal site with no active pyoderma gangrenosum inflammation, cribriform scarring around the periphery at sites of previously active PPG, and a pinpoint papule at the 3 o’clock position (*black arrow*). *PPG*, Peristomal pyoderma gangrenosum.

Four months later, she proceeded with diverting loop ileostomy and bolster placement complicated by PPG flare at the new stomal site. Physical exam revealed erythema and ulcerations at 3 and 9 o’clock positions, along with peristomal skin breakdown and appliance leakage (Fig 1, *A* and *B*). Infectious and intra-abdominal etiologies were excluded. PPG improved with corticosteroids (systemic, topical, and intralesional) and infliximab.

Though her ulcer improved with rapid initiation with oral steroids, she continued to experience intractable, stabbing/burning 10/10 lancing pain localized to a pinpoint papule in the 3 o’clock position (Fig 1, *C*). This pain was reproducible to light touch and refractory to immunosuppressants and systemic analgesics including gabapentin and opioids.

**Question: Which of the following is an appropriate next step in management for this scenario?**

**A.** Increase systemic immunosuppression
**B.** Inject intralesional lidocaine at tender site
**C.** Initiate long-acting opioid therapy
**D.** Begin topical calcineurin inhibitor
**E.** Perform debridement of peristomal tissue

## Answer discussion

**Answer: B.**

Given the resolution of inflammation with persistent, reproducible pinpoint pain, a neuropathic source such as an exposed cutaneous nerve or traumatic neuroma should be considered. Diagnostic administration of intralesional lidocaine is a simple bedside maneuver that can confirm a nerve-mediated pain source when immediate but transient relief occurs.

**Fig 2:**
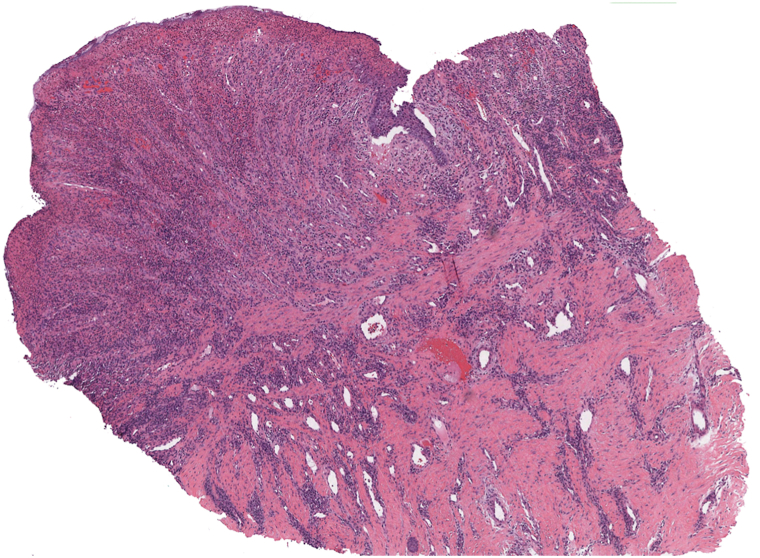
4× magnification of punch biopsy with fibrosis, sparse mixed dermal inflammatory cell infiltrate composed of neutrophils, lymphocytes, plasma cells, and histiocytes, and epidermal hyperplasia, which are common features adjacent to chronic ulcers.

For this patient, test administration of 0.1 mL intralesional lidocaine directly into the lesion resulted in immediate pain resolution. The return of her pain approximately 3 hours later after the anesthetic wore off further supported a nerve-mediated source. Nerve block with liposomal bupivacaine provided short-lived benefit, and ultimately punch excision under local anesthesia resulted in sustained pain relief and discontinuation of all analgesia. While the histologic features of the excised tissue are nonspecific and consistent with common features seen adjacent to chronic ulcers (Fig 2), the marked response to anesthetics and pain resolution post-excision supports the clinical diagnosis of an exposed cutaneous nerve as the pain source.

Ulcer management, including as with PPG, is complex and requires a multipronged approach including immunosuppression, wound care, and pain control.^1^ The ulcer-associated pain should be monitored over the course of wound healing; persistent or disproportionate pain should prompt consideration for alternate etiologies, such as incompletely controlled underlying pathology (eg, pyoderma gangrenosum reactivation), superimposed infection, or other complications.

A percutaneous nerve/neuroma is a non-neoplastic proliferation of an injured nerve, which can arise as a response to trauma (eg, postsurgical or chronic inflammation) as the skin attempts to heal, causing localized hypersensitivity or allodynia.^2^ Trauma can lead to maladaptive alterations in cutaneous nociceptive signaling, resulting in sensitization and ectopic firing of peripheral nerve endings, leading to exaggerated afferent input to central nervous system pathways.^3^

Early recognition of neuropathic pain in similar patients is critical to improve quality of life and reduce unnecessary systemic immunosuppression and analgesia.

## Conflicts of interest

None disclosed.
